# Epidemiology and Classification of Mastitis

**DOI:** 10.3390/ani10122212

**Published:** 2020-11-26

**Authors:** Maros Cobirka, Vladimir Tancin, Petr Slama

**Affiliations:** 1 Department of Animal Morphology, Physiology and Genetics, Faculty of AgriSciences, Mendel University in Brno, Zemedelska 1/1665, 61300 Brno, Czech Republic; maros.cobirka@mendelu.cz; 2 Department of Veterinary Sciences, Faculty of Agrobiology and Food Resources, Slovak Agriculture University in Nitra, Trieda Andreja Hlinku 2, 94976 Nitra, Slovakia; vladimir.tancin@nppc.sk; 3 NPPC—Research Institute for Animal Production, Hlohovecka 2, 95141 Luzianky, Slovakia

**Keywords:** milk, dairy production, mastitis, somatic cell count, causative agent

## Abstract

**Simple Summary:**

Farmers should focus on milk quality over quantity. However, in some situations, more attention is focused on the amount of milk produced. In the long term, this approach might represent an important economic cost as it leads to increased incidence of mastitis. Mastitis affects herds in all countries and is the most economically burdensome disease encountered by dairy farmers. The current review focuses on the main pathogens that cause this inflammation and their prevalence as well as strategies to prevent their proliferation. We discuss economic loss, with the goal of demonstrating that prevention is always better than disease management.

**Abstract:**

Farmers should focus on milk quality over quantity because milk that contains unsuitable components and/or antibiotic residues, or has a high somatic cell count, cannot be used in food production and thereby results in reduced milk yield. One of the main problems affecting the ultimate milk yield of dairy cows is mastitis. This disease is the most serious economic and health problem associated with dairy cow herds and is a major reason for excessive culling. Therefore, many studies have addressed this problem to further our understanding of the agents causing mastitis and their classification and virulence factors. This review summarizes the current knowledge regarding mastitis prevalence, the characteristics of its main causative agents, and the effects of mastitis on dairy production. The review also intends to provide guidance for future studies by examining external effects influencing dairy production in cows under field conditions.

## 1. Introduction

Dairy products, especially milk, are among the most essential food sources for most of the world’s population. The growing global demand for dairy products is driving the need to increase the average milk yield per cow [1]. Increases in milk yield have resulted from genetic selection as well as improved cow nutrition and management. One of the greatest problems impacting high milk yield is poor udder health, particularly due to mastitis [2].

Mastitis, which manifests as inflammation of the mammary gland, is currently one of the most widespread diseases affecting dairy cattle [3,4,5]. Approximately 60–70% of all antimicrobials administered on dairy farms are for preventing and treating mastitis [6]. Mastitis causes a sharp decrease in milk production and farm revenue [7,8,9,10,11,12]. Public health is potentially at risk because mastitis may transmit zoonoses and sicknesses associated with food toxins [13,14]. For this reason, the direct consumption of raw milk is not recommended due to the high probability of contamination with microorganisms from the cow, pasture, milking machine, and containers. Hence, milk pasteurization is mandatory for ensuring its safety as well as to prolong its shelf life [15].

Inflammation is defined as a reaction of tissue to injury [16]. In cases of mastitis caused by bacteria, microorganisms such as *Escherichia coli*, *Streptococcus uberis*, and *Staphylococcus aureus* infect the mammary gland [17], and the prevalence of specific pathogens varies around the world. In some herds, the most serious problem is caused by coagulase-negative staphylococci (CNS) [18], which may or may not appear as an issue in other herds. Thus, the listed causative agents in this review may not be the most common in every area and in every herd.

Somatic cell count (SCC) can be used as an indicator of udder health. Cows that are healthy or that have already recovered from mastitis should have an SCC below 200,000 cells/mL, and cows with counts over 400,000 cells/mL should be considered as having an intramammary infection [19].

Mastitis directly affects the technical characteristics and hygienic quality of milk, indirectly altering its intrinsic qualities [4].

An additional current problem is the ability of pathogens to resist antimicrobial agents. Holko et al. [20] demonstrated that approximately 62% of isolated mastitis-causing agents are resistant to at least one antimicrobial agent. In most cases, the isolated strains demonstrated resistance to streptomycin, neomycin, cephalexin, and penicillin. The worst cases were observed in *Str. agalactiae*, where 100% of isolates were able to resist at least one antimicrobial agent (amoxicillin, amoxicillin/calculate, cloxacillin, penicillin, cephalexin, cephalexin/kanamycin, ceftiofur, cefquinome, tetracycline, streptomycin, neomycin, lincomycin, rifaximin, novobiocin, sulfamethoxazole/trimethoprim, and enrofloxacin). Resistance was found in 86% of *Str. uberis* isolates and 79% of *E. coli* isolates.

This review summarizes the current knowledge regarding mastitis prevalence, the characteristics of its main causative agents, and the effects of mastitis on dairy production.

## 2. Prevalence and Classification of Mastitis

There are many ways to classify mastitis. Mastitis cases can be divided on the basis of origin into environmental and contagious. Environmental mastitis is caused by bacterial microorganisms from the surrounding environment, referred to as environmental pathogens, whereas contagious mastitis is due to spread from other infected quarters [21,22,23]. Generally included among the environmental pathogens are *E. coli*, *Klebsiella* (*K*.) *pneumoniae*, *Enterobacter aerogenes*, and *Str. uberis*. Klaas and Zadoks [23] posited that many microorganisms, such as *S. aureus* and *Str. agalactiae*, can be classified as environmental pathogens even if they are usually classified in the literature as causative agents of contagious mastitis. This view is validated by these pathogens being transmitted through multiple routes—not only through contagious milk from infected cows or poor hygiene during milking but also via bedding, feces, urine, and other contaminants. Algae from the genus *Prototheca* are usually classified among the causative agents of environmental mastitis, but according to Jánosi et al. [24] and Osumi et al. [25], it is unclear whether they are contagious or environmental pathogens. Environmental mastitis is caused by microorganisms present in the animal’s surrounding area. These pathogens infect the udder via the teat canal [26]. According to Nemeth et al. [27], feces constitute the main source of environmental pathogens. The teat canal may remain open for one to two hours after milking [28], and between milkings, the teat end is constantly exposed to environmental pathogens; this is in contrast to contagious pathogens, which usually affect the teat only during milking [21]. According to Idriss et al. [19], most intramammary infections are established during milking or up to two hours thereafter. The process of microbial contamination of milk before and after udder preparation for milking was described by Tancin et al. [29]. Mastitis controls, such as post-milking disinfection of teat ends and dry cow therapy, are far less effective against environmental mastitis-causing agents, but these controls have been proven to be very effective against contagious pathogens [30,31,32].

According to Sharif et al. [33], the most contagious pathogens causing intramammary inflammation are *S. aureus*, *Str. agalactiae*, and *Str. uberis*. Similar data were reported for conditions in Slovakia [19,20,34]. The main reservoirs of contagious pathogens are the rectal, rumen, and genital areas in addition to the mammary gland. The infection is spread during milking time when infected milk contacts an uninfected mammary gland and bacteria then penetrate the teat canal [35]. The prevalence of contagious mastitis can be reduced through the implementation of a five-point program [36], which was later extended to a ten-point program [37]. This program covers aspects of udder health, milking procedures, clinical mastitis therapy, dry cow management, and other management practices to control all mastitis pathogens.

Mastitis can also be classified as clinical or subclinical intramammary inflammation according to symptoms. Clinical mastitis is characterized by sudden onset with redness and swelling of the udder [38]. Milk of an affected quarter is altered, containing flakes or clots and/or has a watery consistency. Cows may be visibly lethargic, have a poor appetite, and usually have fever. Somatic cell count is higher in contrast to the generally normal counts of under 200,000 cells/mL. Subclinical mastitis, by contrast, is characterized by a lack of visible signs in the milk or in the udder [39]. It nevertheless results in decreased milk production (albeit not so much as in clinical cases) and a rise in SCC [40,41]. As reported by Shearer and Harris [42] and Seegers et al. [43], subclinical mastitis occurs 15 to 40 times more often than the clinical form, and its duration is longer. Subclinical mastitis is therefore more difficult to detect, and infection serves as a reservoir of pathogens that spreads the udder infection among animals within the herd.

In recent years, and especially in countries with more highly developed dairy farming industries, the prevalence of contagious mastitis has considerably declined while the relative incidence of environmental mastitis has increased [44,45]. In Table 1, we present data from various studies of pathogen prevalence in differently developed countries. A possible reason for this change is the constant improvement in the levels of hygiene and mastitis control programs on farms, whereas the risk of surrounding area conditions cannot be effectively eliminated. The situation in developing countries is different. Culling of animals infected with mastitis, mostly of the subclinical form, is not practiced in less-developed parts of the world, such as Ethiopia, Uganda, Rwanda, and Pakistan. Hence, the pathogens found in cases of subclinical mastitis usually originate from persistent or chronic infections. The usual source of infections consists of udders affected by pathogens [46]. The disease is mostly transmitted because of poor hygiene during milking and because infected cows are not milked last. As such, cows that are already infected constitute the main source of mastitis in these countries, and pathogens originating in the surrounding environment are of secondary importance [46].

## 3. Major Pathogens Causing Mastitis

In addition to origin-based classification of mastitis-causing agents, they can be divided into major and minor pathogens according to their prevalence and the severity of symptoms. According to Heikkilä et al. [49] and Saidani et al. [50], the major pathogens that cause mastitis are *E. coli*, *S. aureus*, and streptococci. *E. coli* and *S. aureus* can also be transmitted to humans because they are zoonotic pathogens [51]. In Table 2, we present data from various studies.

### 3.1. Escherichia coli

One of the most important pathogens that cause environmental mastitis is *E. coli*. It usually attacks the mammary gland during early lactation, occasionally resulting in lethal consequences if left untreated [55]. Hyperacute *E. coli* mastitis is considered the most common cause of fatal cases [56]. The clinical outcome of *E. coli* mastitis depends upon the severity of infection [57], stage of lactation [55], energy balance [58], vitamin deficiency [54], and vaccination status [55].

When *E. coli* occurs in the mild form, cows show only local signs in the udder and milk, and the duration of symptoms is short. In other more acute cases, it can have very severe or even lethal consequences [57]. The pathogen generally does not invade the alveoli and mammary tissue but, rather, remains in the teat canal and lactiferous sinus. Accordingly, more frequent milking during coliform inflammation is a helpful remedy for the mammary gland [55]. More frequent milking, however, has not been proven to be effective in cases of moderate and severe mastitis, which indicates that the bacteria replicate too quickly for this method to be effective, and that the pathogen has spread to the gland cistern of the udder [59].

The primary cellular defense of the mammary gland against mastitis is phagocytosis, which is mediated by neutrophils [60]. The level, speed, and efficiency of the neutrophil response strongly impact the peak bacterial numbers in the udder and the severity of the disease. This may explain why vaccination against *E. coli* mastitis is more successful than vaccination against other mastitis-causing agents [61]. The two most important factors limiting coliform survival in the udder are the pathogens’ abilities to use lactose and to survive in near-anaerobic conditions. In dry cow mammary glands, iron can be a factor limiting *E. coli* proliferation. During involution through to colostrogenesis, the dry cow udder has a high level of the iron-binding protein lactoferrin, which provides an antibacterial function. If infection is established and if the neutrophil reaction is delayed, *E. coli* can double its population every 20 min [62]. When metabolizing the milk carbohydrate lactose and if the immune system response is weak, the population of colony-forming units of *E. coli* can be as high as 10^8^ per milliliter of milk [63]. The frequency of pathogen distribution within milk samples that tested positive was described in Idriss et al. [19].

The exterior cell wall of *E. coli* contains an endotoxin that has infective potential and plays an important role during the bacteria’s pathogenesis. The endotoxin is regarded as the primary virulence factor of Gram-negative bacteria and is responsible for damage to the udder tissue, but its presence in the mammary gland also evokes leucocyte activity [64]. Hogan and Smith [63] stated that occurrence of new environmental inflammation in the mammary gland is more frequent during the dry period than during lactation. They reported that the highest risk of new mastitis infection occurs during the first two weeks after drying off and in the last two weeks of gravidity. Inflammation arising during the final two weeks prior to calving is particularly dangerous because it almost always persists until lactation [21]. This infection usually starts in the subclinical form and can then develop into clinical mastitis during the early lactation stage, which continues for more than 100 days into the milking period [65]. Smith et al. [21] showed that 65% of clinical mastitis cases occurring during the first two months of lactation originated during the dry period. In a more recent study, Bradley and Green [65] showed that 50% of clinical mastitis caused by *E. coli* originated during the dry period. Therefore, effective dry cow management should be considered an important part of any mastitis control program [66].

### 3.2. Klebsiella pneumoniae

Although generally considered an environmental agent that is mostly present in and transmitted via the surrounding environment, *K. pneumoniae* may occasionally spread from an infected to a healthy cow [61]. It is most commonly prevalent in bedding, particularly in sawdust and peat, which are primary reservoirs of this pathogen [67]. Water and soil are also possible media within which this microorganism can survive and thrive [68,69].

According to Todhunter et al. [52], *K. pneumoniae* is more capable than most *E. coli* strains in overcoming the inhibitory effects of lactoferrin and infecting the mammary gland. As is true of *E. coli* infection, *K. pneumoniae* infection may commence at the end of the dry period as subclinical mastitis and then usually progresses into the clinical form during the start of lactation [65]. As it is a Gram-negative bacterium similar to *E. coli*, the outer layer of its cell wall contains lipopolysaccharides, which are known endotoxins. As mentioned above, this endotoxin is a primary virulence factor that activates cytokines, resulting in damage to mammary gland tissue.

The pathophysiology of intramammary inflammation caused by *K. pneumoniae* has not been studied as extensively as that of *E. coli* mastitis, but a recent review was dedicated to pathogenicity that compared the immune response of cows with these pathogens [70]. The severity of *Klebsiella* spp. mastitis is usually at the mild clinical or subclinical level [71]. In some cases, it may have lethal consequences, as described by Ribeiro et al. [69]. After necropsy of a cow that had been euthanized due to severe symptoms of clinical mastitis caused by *K. pneumoniae*, they found that the microorganism could be cultured from fragments of mammary gland and lungs. The isolate showed the same antimicrobial profile as the strain isolated from the milk of that same cow. This indicated that under some conditions, the pathogen can invade other organs as well.

Milk production losses due to *K. pneumoniae* mastitis are substantial. Concurrent findings in Wilson et al. [72] and Hertl et al. [73] suggest that multiparous cows infected with clinical mastitis lose approximately 4.9 kg of milk per day compared with unaffected cows. In an experiment conducted by Bannerman et al. [74], a test herd was experimentally infected with *K. pneumoniae*. The daily milk yield fell by about 60% on the first day after this intervention. By the second day, the yield dropped to about 15% of the control herd’s yield and, in general, the yield had not recovered by the end of the study.

### 3.3. Streptococcus uberis

According to studies by Zadoks et al. [75] and Davies et al. [76], the general view that *Str. uberis* is an environmental causative agent is debatable, and transmission from cow to cow may be the predominant pathway through which the pathogen is spread. Most authors have categorized *Str. uberis*, which originates in the animals’ surroundings, as an environmental causative agent. Similar to *K. pneumoniae*, *Str. uberis* is mostly present in bedding materials such as peat or straw [67]. This pathogen can also be found on the bodies of animals, such as on the outer skin of the udder or the muzzle. This shows that reciprocal sucking of other infected cows can be one of the pathways through which *Str. uberis* is transmitted from infected to healthy cows [77]. The original infection usually occurs between milkings, so disinfection, cleaning, renewing of bedding material, and manure removal help with controlling the environmental mastitis associated with this pathogen. In pasture-based dairy farming systems, the pasture itself is a primary reservoir of *Str. uberis* [78].

Traditional bedding materials, such as straw, can be rather expensive and their availability insufficient. As a result, recycled manure solids that are physically separated from slurry are sometimes used as bedding material. This has led to the increasing prevalence of environmental mastitis in recent years [79].

The severity of mammary gland inflammation depends upon the host immune system reaction, type of pathogen, and the specific strain as some particular strains are more infectious than others [80]. The majority of *Str. uberis* infections occur in the dry period and are usually of subclinical form [81]. These infections commonly develop into acute cases during subsequent lactation [82]. According to Wilkinson [83], 56% of all mastitis diagnosed during the lactating period originated in the dry period. Denis et al. [84] found that macrophages from cows in the dry period are more active than those from mid-lactation cows, even though *Str. uberis* infection usually occurs during the dry period. The rates of recovery from these infections were shown to be higher among first- and second-parity cows compared to older cows [85].

As early as six days after the infection is established, this pathogen begins to damage the alveolar tissue (causing fibrosis), similar to *S. aureus*. This may explain why *Str. uberis* has been observed to respond poorly to antibiotic treatment [86]. Conversely, Pyörälä [87] reported that mastitis caused by environmental causative agents such as *E. coli* or *Str. uberis* can, in most cases, be cured by antibiotic therapy.

The duration of mastitis cases caused by this pathogen varies widely and cannot be generalized. Even though most such infections last relatively short periods ranging from 16 to 46 days [88,89], cases lasting markedly longer (from 2 to 20 months) have also been reported [90,91]. Particularly in persistent cases, an increased chance of reinfection has been observed after *Str. uberis* infections of the mammary gland have been successfully remedied. A similar reinfection risk has been observed in cases of *S. aureus* mastitis [92].

### 3.4. Streptococcus agalactiae

According to Lyhs et al. [93], several sources of indirect evidence suggest that milking personnel may transmit *Str. agalactiae* into cattle herds. A study characterizing *Str. agalactiae* strains isolated from udder mastitis and from human infections were shown to have 58% similarity, and clustering of the isolates showed they shared 70% genetic similarity [94]. Infections from human strains are more likely to spontaneously heal than those originating from strains infecting udders of other animals [95]. The self-cure rate is very low in animal-to-animal transmitted strains [72].

Indirect evidence shows that younger cows in their first lactation period are more resistant to contagious causative agents [96]. The pathogen can survive for a relatively long time and persist, undetected, in the udder. These animals serve as infection reservoirs and sources for spreading the disease [97].

Unlike *S. aureus* and other contagious pathogens, *Str. agalactiae* cannot multiply or grow outside the udder but can survive for a short duration on the hands of milking personnel, milking machines, and teat surfaces. This may be sufficient for its spread to healthy cows during milking. Even if herd hygiene is adequate, some risk is associated with buying new cows if they are infected and not held in quarantine before integration into the herd. *Str. agalactiae* is known for its high infectivity, rapid spread, and silent nature [98].

The prevalence of *Str. agalactiae* demonstrates that this bacterium is a significant cause of mastitis, especially in herds that are not well managed and have poor hygiene. Studies by Tolla [99], Sharif et al. [33], Kassa et al. [100], and Lakew et al. [101] have shown that the main causative agents of mastitis in less-developed countries such as Ethiopia or Pakistan are the contagious ones and that the most prevalent mastitis cases are those of contagious origin. This could be associated with unhygienic milking practices and poor herd management. Conversely, Tomazi et al. [102] stated that the prevalence of *Str. agalactiae* has become very low in particular regions of Europe and North America, mainly due to improved mastitis control programs. This pathogen responds well to antibiotic therapy and can be eradicated from dairy herds with good mastitis control practices, such as teat dipping after milking and dry period therapy [40].

Blitz therapy is one of the approaches most widely used to eradicate *Str. agalactiae* mastitis. This method involves simultaneously treating all cows in the herd, regardless of infection status. However, this procedure is expensive and may create antimicrobial resistance. The method is usually modified so that only those animals testing positive for infection are treated, particularly because preventive use of antibiotics is prohibited in some countries [103].

### 3.5. Staphylococcus aureus

Khan and Khan [40] stated that infections caused by *S. aureus* remained the largest mastitis problem in dairy cattle because the cure rate using antibiotics is very low during lactation, and, in many cases, the infections become chronic, making culling of the affected animal frequently necessary. Mastitis caused by this pathogen is only successfully controlled through preventing new infections and the culling of affected animals. Similar to other contagious pathogens, it is spread via milking machine components, the hands of milking personnel, and through washcloths [35].

Even though *S. aureus* strains are susceptible to a wide variety of antibiotics in vitro, farmers often complain that cure rates under in vivo conditions are lower than expected. Possibly confirming this observation is evidence that *S. aureus* has a strong ability to survive under neutrophil activity [104], inducing fibrosis in the udder and invading mammary epithelial cells [105]. The primary reason for the low cure rate, however, lies in its ability to create microabscesses that prevent antibiotics from reaching the pathogen [35,106]. According to research findings, the production losses caused by staphylococcus mastitis are usually long term in nature. The pathogen causes permanent damage to the udder’s secretory tissue, which is subsequently displaced by non-secretory tissue, and this diminishes the cow’s milk-producing ability [107].

Zadoks et al. [54] stated that even though *S. aureus* can be transferred from cow to cow, it can also remain in a given dairy barn environment between milkings. Heifers are proven reservoirs of this pathogen. In Trinidad et al. [108], 12–15% of first-lactation cows were already infected with *S. aureus*. Many of them remained infected during the whole lactation, unnoticed, and served as reservoirs for the spread of the infection to other cows in the herd.

In general, 10–12% of clinical mastitis cases are caused by *S. aureus* [109]. The pathogen’s response to in vivo treatment is poor, and *S. aureus* usually persists in the udder [110]. The best-known resistance is to penicillin antibiotics, but the level of the resistance varies between study years and countries [111,112,113].

Mastitis caused by *S. aureus* is usually subclinical and chronic, and in Tenhagen et al. [114], was shown to occur more often in late than in early lactation. This corresponds with observations of Laevens et al. [115] and Tančin et al. [116], who found that SCC was higher in later-lactation cows. Hertl et al. [73] observed that infection caused by *S. aureus* had a more critical impact on milk production if it occurred during first or second lactation than during the third and subsequent lactations. Partially similar results were reported by Reksen et al. [117]; however, in their study, the largest milk losses occurred during the first and third lactations, but the impacts were lesser during the second lactation.

### 3.6. Streptococcus dysgalactiae

Although Fox and Gay [53] noted that the status of *Str. dysgalactiae* as a contagious or environmental causative agent of mastitis might be debatable, its importance as a mastitis pathogen is clear. Khan and Khan [40] shared this opinion and pointed out that *Str. dysgalactiae* may live almost anywhere: the mammary gland, rumen, feces, bedding, and in the barn. The pathogen has been isolated not only from the udder but also from other body parts of animals, including the muzzle, tonsils, and vagina [77,118]. *Str. dysgalactiae* mastitis may occur during the dry period in herds, even when there have been no previous observations of this particular type of infection [82].

This pathogen is seldom studied separately; studies usually do not distinguish *Str. dysgalactiae* from *Streptococcus* spp. [49]. Its prevalence is often comparable to or even exceeds that of *Str. uberis* [119,120].

The presence of this pathogen in dairy herds is serious because the inflammation caused by this agent is usually acute [121]. Yeruham et al. [122] stated that *Str. dysgalactiae* originates in the surroundings of the animals and can, in rare instances, be transmitted via insect vectors, such as wasps or flies. However, this kind of transmission can only be observed during the summer. The pathogen is capable of neutralizing nonspecific immunity of the animal by secreting enzymes and toxins capable of overcoming this otherwise effective defense [123].

## 4. Host Response and Pathogen Infectivity

The first line of udder defense against pathogens is the teat end. It is open and closed by a sphincter composed of smooth muscles that serves as a barrier to prevent pathogens from entering the canal and prevent milk from escaping. The teat canal is lined with the stratified squamous epithelium, which creates keratin to fill the canal between 30 min and two hours after milking. This time span may vary, creating an opportunity for bacteria near the opening to enter the teat canal [55]. The keratin is composed of fatty acids and fibrous proteins. After pathogens enter the teat canal, the fibrous proteins bind electrostatically to the pathogens, alter their cell walls, and thereby render them more susceptible to osmotic pressure. Inability to maintain osmotic pressure causes lysis of the cell membranes and death of the invading pathogens [55,124]. The teat end’s level of defense against pathogens depends upon several specific physical and physicochemical factors including, among others, teat canal length and width, amount of keratin present, and milkability as measured by peak flow rate [41,42]. Bacterial pathogens able to traverse the canal opening and escape the antibacterial activities of keratin establish the disease process in the mammary gland.

The second line of defense, which is the immune response of the host, must then respond. Burvenich et al. [55] reported that the severity of the disease mostly depends on the immune response and genetic predisposition of the host, but Fernandes et al. [125] stated that the virulence of the bacterial strains may also play a major role in disease severity. Among the main pathogen-related factors are species, virulence, strain, and size of the bacteria inoculum, whereas host factors include parity, stage of lactation, age, immune status of the animal [126], stress [127], and vaccination status [128].

The response of the immune system to the early stages of infection is reliant on cell surface receptors, which can recognize microbial molecules [129], in addition to soluble components, such as acute phase proteins and cytokines, which are secreted into blood and milk [130]. The host response is specific to different types of mastitis-causing agents [131]. According to Hernández-Castellano et al. [132], Gram-negative bacteria, such as *E. coli*, elicit much higher immunoglobulin G concentrations and lactate dehydrogenase activity compared with the Gram-positive bacteria. This means that lactate dehydrogenase in combination with SCC may be used as a marker to differentiate between Gram-positive and -negative bacteria.

Currently, treatment of mastitis mostly involves the use of antibiotics, though glucocorticoids such as prednisolone are added to these formulations [133]. They help the threatened host organism to increase the integrity of the blood–milk barrier and to restore the decreased milk quality caused by mastitis [134]. Prednisolone can bind to the glucocorticoid receptor on cells and block the production of proinflammatory cytokines [129]; more importantly, it can affect the recruitment of immune cells to sites of inflammation. However, prednisolone also impairs the immune system of the mammary gland by reducing the concentration of immunoglobulin G in the milk; therefore, it could have both helpful and harmful effects on mastitis treatment [134]. Wall et al. [135] found that supraphysiological doses of oxytocin open the blood–milk barrier and enhance the opening during lipopolysaccharide-induced mastitis. These findings mean that oxytocin induces a greater transfer of the blood component to milk and can be effectively used in cases where there is little or no transfer of immunoglobulin G. This usually occurs during subclinical cases of mastitis.

To establish inflammation, a pathogen must not only enter the udder but also be able to survive and multiply to produce a pathogenic effect [50]. The causative agents of mastitis vary in their virulence [125], just as animals vary in their resistance to microbial entry into the mammary gland and their subsequent response to overcoming inflammation [126].

## 5. Effect on Dairy Production

Substantial losses in milk production occur as a result of both clinical and subclinical mastitis [136]. St. Rose et al. [137] found that milk production does not improve even after complete recovery from subclinical mastitis, so the economic loss is still substantial. Although antibiotic treatment prevented subclinical mastitis from progressing to the clinical form during lactation, the subclinical form endured even after treatment.

Berry et al. [138] estimated the cost of an average case of mastitis affecting a dairy cow with a production of 7000 kg of milk per lactation as approximately GBP 131 (EUR 198). This value includes labor, treatment, drugs, veterinary charges, discarded milk, milk production loss, feed intake needed for production of discarded milk, and occasional fatality of the disease. In Bar et al. [139], this cost was determined as USD 179 (EUR 162), while Cha et al. [140] reported a cost of USD 155 (EUR 140) and Dahl et al. [141] reported USD 148 (EUR 134). These numbers mostly depend on the actual price of milk and medication as well as the severity and duration of the disease. The lactation length of each infected animal is shortened by approximately 57 days according to Khan and Khan [40], and Seegers et al. [43] reported average milk losses of 375 kg per lactation.

The milk from an infected mammary gland is affected in many ways, including changes to its composition. The losses of fat, casein, and lactose are significant [142,143]. According to statistics from the National Mastitis Council [37], in the USA, losses due to reduced production as a result of mastitis plus prevention and control costs exceeded USD 2 billion annually and approximately one-third of all cows were affected.

A pronounced difference exists between milk losses in cases of clinical versus subclinical mastitis. Some researchers observed no significant effect on milk losses in cases of subclinical quarter intramammary infection by minor pathogens [144,145,146]. Gonçalves et al. [146] found no significant changes in the concentrations of protein or fat between healthy cows and cows affected by subclinical mastitis caused by miscellaneous pathogens. These cows were found to have only moderately higher SCC but with no notable impact on milk yield. By contrast, clinical mastitis can cause direct and tremendous losses in milk. Bezman et al. [10] found that quarter milk yield decreased by 20% in cases of *Str. dysgalactiae* mastitis and by 50% in cases of *E. coli* mastitis.

Milk produced by a mammary gland affected by clinical mastitis is useless in dairy production and should be discarded. Economic losses due to discarded milk are much greater than losses due to the diminished milk production because the discarded milk is produced by cows and, thus, has incurred the associated feeding costs that are accounted for in the loss. This means that economic loss due to 10 kg of discarded milk is greater than the loss for 10 kg of decreased milk production. To reduce economic losses, this discarded milk is often fed to calves instead of milk replacer, but only under certain milk conditions, and thus reduces the costs of replacer [4]. However, Heikkilä et al. [9] reported that decreased milk production is a long-term loss, which is the why it causes greater financial loss than milk discarded in the short term.

According to Halasa et al. [4] and Gussmann et al. [147], economic losses related to mastitis can be divided into those associated with the treatment of ill animals, losses caused by increased mortality due to infection and culling of afflicted animals, and indirect losses due to reduced milk production. Hogeveen et al. [148] further subdivided the economic losses, separating the economic costs of mastitis into preventive and failure costs. Preventive costs are associated with the resources needed to sustain a mastitis prevention and control program for healthy animals. Failure costs include the resources needed to resolve problems in animals already affected by mastitis, such as antibiotics, treatment, veterinary work, etc. Failure costs are aligned with milk production loss, but only a few authors [149,150,151] have similarly classified the economic consequences of mastitis.

## 6. Conclusions

In producing milk, quality must be more important than quantity. The food industry cannot process milk with a high SCC or milk containing antibiotic residues. This means that controlling mastitis is essential. Driving this heightened concern are that mastitis remains the costliest medical and economic problem in the milk-producing industry and that pressure is increasing to avoid use of antibiotics. Dairy farmers should focus on preventing mastitis infections while implementing and adhering to a mastitis control program. The reality is often quite different, however, and farmers may wait until after mastitis appears to begin resolving the problem. Although mastitis control programs are key to protection from and prevention against mastitis, their successful implementation depends upon the farmer’s comprehensive knowledge and the correct classification of mastitis-causing agents. Determining the origin and transmission of many pathogens in field conditions is often difficult (e.g., distinguishing between *S. aureus* and *Str. uberis*, both of which can be transmitted in multiple ways). The results from numerous studies vary, even for the main causative pathogens. Nevertheless, the prevalence of clinical and acute mastitis cases in a herd may be reduced to minimal levels through constantly observing and improving hygienic practices during milking and proper treatment of milking cows, effective dry cow therapy management, and prudent medication of cows with subclinical and chronic mastitis. Additionally, cows with continuously increasing SCC and without response to treatment should be culled from the herd. Consistent efforts to reduce the use of antibiotics and the application of new approaches in dairy herds may also help to prevent and cure this disease through better understanding of the involved environmental factors and their implementation into the managing of dairy herds. For this purpose, it is important to develop new immunotherapies that do not involve the use of antibiotics. For instance, strategies aimed at better fulfilling immune cell potential and the use of natural immunomodulators as cytokines for regulation of mammary gland inflammation.

## Figures and Tables

**Table 1:** Prevalence of pathogens isolated from milk of dairy cows in percentages.

Pathogen	USA	Slovakia	Ethiopia
*E. coli*	9.1	14.8	13.6
*K. pneumoniae*	3	<1.0	-
*Str. uberis*	24.9	10.9	-
*Str. agalactiae*	-	5.8	-
*Str. dysgalactiae*	7.8	<1.0	-
*S. aureus*	1.3	12.5	39
*Enterococcus* spp.	1.3	5.2	-
*Streptococcus* spp.	10.4	1.5	20.3
*Staphylococcus* spp.	5.2	-	-
CNS	-	35.9	18.6

Note: Table modified according to Ganda et al. [47], Asmare and Kassa [48], and Holko et al. [20].

**Table 2:** Classification of mastitis-causing pathogens.

Source	Mammary Gland	Environment
Major pathogens	*S. aureus* *Str. agalactiae* (*Str. dysgalactiae*) *Mycoplasma bovis*	Environmental streptococci: *Str. uberis, Str. equinus, (Str. bovis)*, (*Str. dysgalactiae*) *Enterococcus* spp.: *E. faecalis*, *E*. *faecium*, *E*. *durans* Coliforms: *E. coli*, *K*. *pneumoniae*, *K*. *oxytoca*, *Enterobacter aerogenes* Non-coliforms: *Proteus* spp., *Serratia* spp., *Yersinia* spp. Others: *Pseudomonas aeruginosa*, *Arcanobacterium pyogenes*
Minor pathogens	CNS: *S. chromogenes*, *S. haemolyticus*, *S. epidermidis*, *S. simulans*, *S. sciuri* *Corynebacterium bovis*	*Yeasts* *Fungi*

Note: Table modified according to Smith et al. [21], Todhunter et al. [52], Fox and Gay [53], Zadoks et al. [54], Heikkilä et al. [49], and Saidani et al. [50].
